# *Mycobacterium tuberculosis* Rv1152 is a Novel GntR Family Transcriptional Regulator Involved in Intrinsic Vancomycin Resistance and is a Potential Vancomycin Adjuvant Target

**DOI:** 10.1038/srep28002

**Published:** 2016-06-28

**Authors:** Jie Zeng, Wanyan Deng, Wenmin Yang, Hongping Luo, Xiangke Duan, Longxiang Xie, Ping Li, Rui Wang, Tiwei Fu, Abualgasim Elgaili Abdalla, Jianping Xie

**Affiliations:** 1 Institute of Modern Biopharmaceuticals, State Key Laboratory Breeding Base of Eco-Enviroment and Bio-Resource of the Three Gorges Area, Key Laboratory of Eco-environments in Three Gorges Reservoir Region, Ministry of Education, School of Life Sciences, Southwest University, Beibei, Chongqing 400715, China; 2 Department of Clinical Microbiology, College of Medical Laboratory Sciences, Omdurman Islamic University, Omdurman, Khartoum, Sudan

## Abstract

Novel factors involved in *Mycobacteria* antibiotics resistance are crucial for better targets to combat the ever-increasing drug resistant strains. *Mycobacterium tuberculosis* Rv1152, a novel GntR family transcriptional regulator and a promising vancomycin adjuvant target, was firstly characterized in our study. Overexpression of Rv1152 in *Mycobacterium smegmatis* decreased bacterial susceptibility to vancomycin. Moreover, a deficiency in MSMEG_5174, an Rv1152 homolog made *M. smegmatis* more sensitive to vancomycin, which was reverted by complementing the MSMEG_5174 deficiency with Rv1152 of *M. tuberculosis*. Rv1152 negatively regulated four vancomycin responsive genes, namely genes encoding the ribosome binding protein Hsp, small unit of sulfate adenylyltransferase CysD, L-lysine-epsilon aminotransferase Lat, and protease HtpX. Taken together, Rv1152 controls the expression of genes required for the susceptibility to vancomycin. This is the first report that links the GntR family transcriptional factor with vancomycin susceptibility. Inhibitors of Rv1152 might be ideal vancomycin adjuvants for controlling multi-drug resistant Mycobacterial infections.

Tuberculosis, caused by *Mycobacterium tuberculosis* (*M. tuberculosis*) infection, remains the second highest pandemic disease with formidable rate of morbidity and mortality worldwide^1^, particularly in developing countries and HIV co-infected population despite decade’s implementation of TB control programs^2^. The emergence of multidrug-resistant strains of *M. tuberculosis*^3^, extensively drug resistance (XDR), even totally drug resistance (TDR) cases of TB^4^,^5^,^6^ is worsening the TB control. Globally, an estimated 480,000 cases of multidrug-resistant TB (MDR-TB) was reported in 2013. The genus *Mycobacterium* includes both pathogenic and saprophytic species that are able to survive under environmental stresses, including oxidative and genotoxic stress, hypoxia, nutrient starvation and multiple antibiotics^7^,^8^. Transcriptional regulation plays an important role in the bacterial response to environmental stresses. The GntR family of bacterial regulators is named after the *Bacillus subtilis* transcription regulator GntR, the first characterized transcriptional GntR-type repressor required for gluconate metabolism^9^,^10^. This holds true for the GntR family of transcriptional regulators, with around 2000 members in both bacterial and archaea genomes^9^,^11^. The proteins belong to GntR family share a characteristic conserved N-terminal domain with winged helix-turn-helix that is involved in DNA binding, which can be easily recognized by a Conserved Domain Database (CDD) search^12^. GntR consists of six subfamilies differing in C-terminal signaling domains involved in the effector binding^11^, namely FadR, HutC, MocR, YtrA, AraR and PlmA^13^,^14^. GntR regulators are defined as a part of specific subfamily^15^. The structures of FadR alone and in complex with its effector and operator DNA have been recently determined^16^,^17^,^18^,^19^, before no structural information is available for other three subfamilies of GntR-like regulators. GntR regulators in *M. tuberculosis*^20^ and *M. smegmatis*^21^ are just to only bioinformatically predicted, while lacking experimental evidences for the proposed functions.

In this study, we identified a novel *M. tuberculosi*s GntR family regulator, Rv1152, which can alter cell wall permeability of *M. smegmatis* to acid and surface stress and play an important in vancomycin loss of susceptibility through negatively regulating the genes responsive to vancomycin. In brief, *M. smegmatis* overexpressed *M. tuberculosis* Rv1152 (MS_Rv1152) was more resistant to vancomycin than *M. smegmatis* harboring the vector only (MS_Vec), while the MSMEG_5174 (the homologous gene of Rv1152 in *M. smegmatis*) deletion mutant (△MSMEG_5174) was more sensitive to vancomycin than the wild type *M. smegmatis.* More importantly, the susceptibility phenotype of △MSMEG_5174 to vancomycin can be complemented by the Rv1152 (△MSMEG_5174::Rv1152). Several vancomycin responsive genes were down regulated in *M. smegmatis* overexpressed Rv1152 strain, while the expression of the same set of vancomycin responsive genes was up regulated in homologous gene MSMEG_5174 knock out strains. The genes regulated by Rv1152 are responsible for the sensitivity of *M. smegmatis* to vancomycin. These data suggest that Rv1152 involved in the loss of susceptibility to vancomycin through negatively regulating the expression of vancomycin responsive genes.

## Material and Methods

### Strains, Plasmids and Primers

*M. smegmatis* mc^2^155 strains were preserved by the Institute of Modern Biopharmaceuticals. *Escherichia coli* DH5α strain used for gene clone was grown at 37 °C in Luria-Bertani (LB) broth or on LB agar with appropriate antibiotics. *M. smegmatis* was grown at 37 °C in Middlebrook (MB) 7H9 liquid medium or on MB 7H10 agar supplemented with 0.2% (w/v) glucose, 0.5% (v/v) glycerol and 0.05% (v/v) Tween 80. Hygromycin (100 μg/ml) was added when required. All strains were stored with sterile 20% glycerol at −80 °C for further use. The genomic DNA of *M. tuberculosis* H37Rv was provided by Chongqing Pulmonary Hospital. The bacterial strains and plasmids used in this study are described in Table 1. All the PCR primers were synthesized by BGI (Shenzhen, Guangdong, China) and the sequences of primers are listed in Table 2.

### Construction of overexpression strains

The full length of Rv1152 gene and several vancomycin responsive genes was amplified from *M. tuberculosis* H37Rv genome DNA using the specific gene primer pairs (Table 2). For Rv1152, The PCR product and the plasmid pALACE were digested with *BamH*I and *Cla*I to generate the recombinant pALACE-Rv1152. For the vancomycin responsive genes in *M. tuberculosis* and their homologous genes in *M. smegmatis*: including Rv0251c (MSMEG_0424), Rv1285 (MSMEG_4979), Rv0563 (MSMEG_1134) and Rv3290c (MSMEG_1764), the PCR products were ligated into the plasmid pALACE digested by *BamH* I and *Nde*I. All plasmids were electroporated into *M. smegmatis*, a non-pathogenic, fast-growing mycobacterium that, serves as a surrogate model organism to study genes functions of the virulent *M. tuberculosis*^22^,^23^. The electroporated recombinant *M. smegmatis* strains were plated on Middlebrook (MB) 7H10 agar containing 50 μg/ml hygromycin after *in vitro* growth in MB 7H9 liquid medium for 3 hour. The positive strains were further verified by Western blot.

### Western blot and subcellular localization

Generally, the acetamide-induced recombinant MS_Rv1152 and MS_Vec were sonicated. The whole lysates were centrifuged at the speed of 3,000 × g for 5 min at 4 °C to remove un-lysed cells and cell debris. The supernatants were ultra-centrifuged at the speed of 27,000 × g for 40 min at 4 °C. After ultra-centrifugation, the pellets were considered the cell wall fraction, and the supernatants were supposed to be cell membrane and cytosol fractions. The pellets were further suspended in 1 × PBS. Equal amounts of protein from pellet and supernatant fractions were subjected to Western blotting for analyzing the expression of Rv1152. Native *M. smegmatis* GroEL2, which contains a string of endogenous histidines^24^, was detected as a cytoplasmic control by an anti-His mouse primary antibody (TIANGEN, China).

### Construction of the genetic deletion and complementation strains

An in-frame deletion of the gene MSMEG_5174 (the homolog of *M. tuberculosis* Rv1152), a GntR-family response regulator of *M. smegmatis* was constructed using Xer site-specific recombination^25^. Two DNA fragments, comprising 600 and 700 bp (including the first 50 bp and the last 50 bp of the gene, respectively), were amplified from the genome of *M. tuberculosis* using specific primers (Table 2), and cloned at the borders of the hygromycin excisable cassette. The resulting DNA fragment was purified and introduced by recombineering into *M. smegmatis* derivative containing pJV53, a replicative plasmid expressing two phage recombinases and conferring kanamycin resistance^26^,^27^. Two hygromycin-resistant colonies were isolated and tested by PCR forthe correct integration of the excisable cassette into the chromosome (not shown). Subsequently, they were grown for 10 generations in the absence of hygromycin and kanamycin to allow excision of the hygromycin cassette and the loss of pJV53. Hygromycin and kanamycin-sensitive colonies were finally recovered at the expected frequency. One of them was analyzed in parallel with a colony of the wild-type parental strain by PCR with primers flanking the region used for the recombination. BGI sequencing confirmed the successful construction of the knockout strain *M. smegmatis* (△MSMEG_5174).

For the complementation strains, the Rv1152 complemented *M. smegmatis* strains (△MSMEG_5174::Rv1152) were constructed by integrating *M. tuberculosis* homolog Rv1152 into the chromosomes of the respective deletion strains. Briefly, the Rv1152 gene was first cloned into a pALACE vector, and the recombinant plasmid pALACE_Rv1152 was transformed into the respective *M. smegmatis* mutant strains. The complementation strain was selected on 7H10 medium (complemented with 0.2% glycerol) containing 50 μg/mL hygromycin and the hygromycin-resistant strains were selected and further confirmed by using Western blot.

### *In vitro* growth of the bacteria under acid and surface stresses

Growth patterns of the two recombinant mycobacteria were examined according to previously described procedures^28^. Briefly, *M. smegmatis* was grown overnight in Middlebrook 7H9 medium (complemented with 0.05% Tween 80 and 0.2% glycerol). Recombinant MS_Vec and MS_Rv1152 were grown in presence of surface stress and acidic stress. For surface stress, acetamide-induced MS_Vec and MS_Rv1152 were treated with 0.05% SDS for 1, 2, 3 and 4 h. For acidic stress, HCl was added into the 7H9 medium and adjusted to pH = 4. MS_Rv1152 and MS_Vec were exposed for duration of 3, 6 and 9 h, respectively. After SDS and acidic treatment, the recombinant strains were diluted and plated onto MB 7H10 agar containing hygromycin, the bacteria were counted after 3 days of incubation.

### The MIC determinations for antibiotics

Seven antibiotics including vancomycin (Van), norfloxacin (Nor), ciprofloxacin (Cip), ofloxacin (OFL), erythromycin (Ery), isoniazid (INH), and rifampicin (Rif) were used in this study. Growth patterns of the wild type *M. smegmatis* strain (WT), the gene deletion mutant (△MSMEG_5174), and complementation strains (△MSMEG_5174::Rv1152), the overexpression strain (MS_Rv1152) and the control strain (MS_Vec) were measured according to the procedures described previously with minor modification^23^,^29^. The MICs of antibiotics were determined by using serial two-fold dilution of the antibiotics in 7H9 medium as previously described^30^. Briefly, wells of a 96 well microtiter plate were filled with 100 μl of 7H9 medium. The required highest antibiotic concentration was prepared and 200 μl were added to the first vial. This was serially diluted to halve the concentration by mixing with equal volume of bacterial culture in the subsequent wells till second to the last well. Last vial was the control without antibiotics. The *M. smegmatis* strains were grown in replicates in 7H9 medium to an OD_600_ of 0.8, 1% of original bacteria was inoculated to 100 μl of the prepared culture with or without antibiotics. MIC values of each antibiotic were determined as drug concentration that inhibited bacterial growth by at least 99%.

### Determination of bactericidal effect of antibiotics

To determine mycobacterial growth curves and the effect of antibiotics, *M. smegmatis* strains including WT, MS_Vec, MS_Rv1152, △MSMEG_5174, and △MSMEG_5174::Rv1152 were grown overnight in Middlebrook 7H9 broth (supplemented with 0.05% Tween80 and 0.2% glycerol). Hygromycin was not added in the 7H9 medium when assaying antibiotics resistance of all strains. When cells entered a stationary growth phase (OD_600_ between 0.8–1.0), each culture was 100-fold diluted in 100 μl of fresh 7H9 broth containing the indicated concentration of each antibiotic. The cultures were then allowed to grow further at 37 °C with shaking at 110 rpm. After 24 h treatment with these antibiotics with different concentration, the bacteria were diluted by 10-fold and plated into 7H10 agar medium. The bacterial numbers were counted after 3 days culture. The medium without any antibiotics serves as the control to make sure the normal growth of bacteria.

### RT-PCR detection of the gene transcription

Isolation of mRNA and cDNA preparation were performed from wild type strains (WT), MSMEG_5174 deletion mutants (△MSMEG_5174), Rv1152 complementation strains (△MSMEG_5174::Rv1152), *M. smegmatis* harboring pALACE (MS_Vec) and Rv1152 overexpression *M. smegmatis* strains (MS_Rv1152). RT-PCR was used to compare the transcriptional levels of genes expression using gene specific primers and the real-time PCR analysis was subsequently carried out according to previously described procedures^31^. The reactions were performed in a RT-PCR machine (Bio-Rad IQ5) under the following thermocycling parameters: 95 °C for 5 min and 40 cycles at 95 °C for 30 s, 60 °C for 30 s and 72 °C for 30 s. Amplification specificity was assessed using melting curve analysis. Gene expression levels were normalized to the levels of *sigA* gene transcription. Average relative expression levels and standard deviations were determined from three independent experiments. All the gene specific primers used for RT-PCR were listed in the Table 1S.

### Statistical analysis

The experiments were performed in triplicate. Differences between groups were analyzed by using Prism 6 and Student’s t test. ***P < 0.001, **P < 0.01, *P < 0.05, means ± SEM from at least three biological replicates.

## Results

### *M. smegmatis* overexpressing Rv1152 showed altered response to stresses

**Figure 1:**
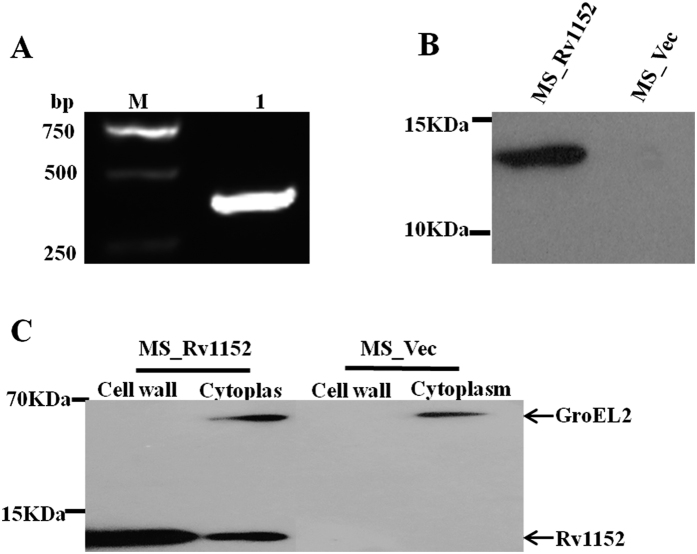
Over-expression of Rv1152 in *M. smegmatis*. (**A**) PCR amplification of Rv1152 gene from *M. tuberculosis*. (**B**) Western blot demonstrated the expression of His-tagged Rv1152 protein in *M. smegmatis*. (**C**) Rv1152 is associated with cell wall and cytoplasm of *M. smegmatis*.

To study the function of Rv1152, we constructed the recombinant *M. smegmatis* harboring pALACE_Rv1152 (MS_Rv1152) and vector only (MS_Vec). Rv1152 gene was successfully amplified from the *M. tuberculosis* genome by using gene specific primers (Fig. 1A). Western blot showed the presence of the expressed 14 kDa his-tagged in MS_Rv1152 but not in MS_Vec (Fig. 1B), suggesting that Rv1152 was successfully expressed in *M. smegmatis*. Rv1152 localized to both cell wall and cytoplasm of *M. smegmatis*: the target proteins were found in cell wall and cytoplasm of MS_Rv1152, but not in MS_Vec while the cytoplasm marker GroEL2 of *M. smegmatis* was detected in both MS_Rv1152 and MS_Vec (Fig. 1C).

**Figure 2:**
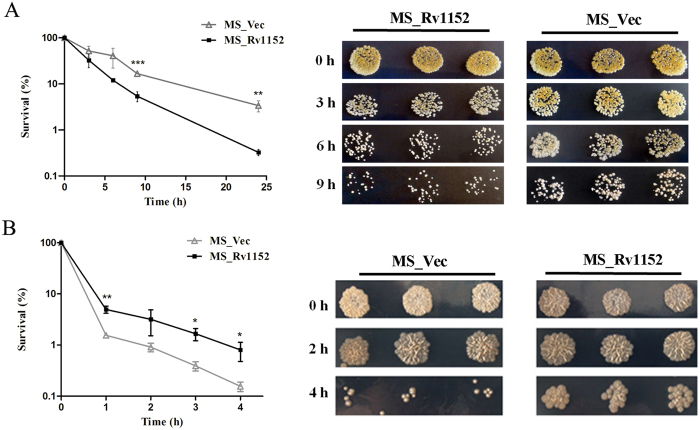
Effect of Rv1152 overexpression on acid and surface stress tolerance. (**A**) Survival of recombinant MS_Vec and MS_Rv1152 after 0, 3, 6 and 9 h treatment with low pH (pH = 4). (**B**) Survival of recombinant MS_Vec and MS_Rv1152c after exposure to 0.05% sodium dodecyl sulfonate (SDS) for 0, 1, 2, and 4 hours.

The ability of *M. tuberculosis* to survive within the host requires resistance to various physiological and environmental stresses. Within granuloma, *M. tuberculosis* is able to persist for years even under severe stresses such as hypoxia, nutrient limitation, reactive oxygen and nitrogen intermediates, low pH, alveolar surfactants, and free fatty acids^32^. However, bacterial factors that enhanced their survival under hash condition remain poorly understood. Mycobacterial responses to cell surface stress are of particular interest for understanding the pathogenesis of *M. tuberculosis* and its susceptibility to antibiotics^33^. The recombinant MS_Rv1152 showed more cell death than MS_Vec when the bacteria were exposed to acid stress (Fig. 2A). Although there was a rapid decrease in the bacterial numbers for all tested strains exposed to the detergent SDS, in comparison with MS_Vec, MS_Rv1152 was more tolerant to SDS: the percentage survival values were 5% for the MS_Rv1152 and 1.5% for the MS_Vec. The greater tolerant of the MS_Rv1152 to SDS was verified when bacterial survival was tested after 1 hour when incubation with SDS (Fig. 2B). These results suggest that the overexpression of Rv1152 in *M. smegmatis* modified the *M. smegmatis* response to surface and acid stress.

### Rv1152 confers *M. smegmatis* reduced susceptibility to vancomycin

**Figure 3:**
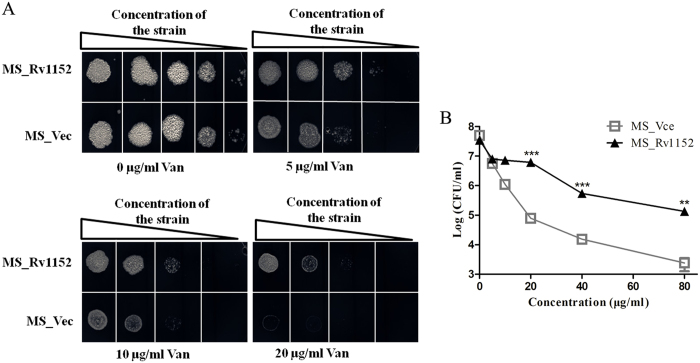
Overexpression of Rv1152 in M. smegmatis decreases vancomycoin susceptibility. (**A**) Ten-fold serial dilutions of MS_Rv1152 and MS_Vec were plated onto 7H10 agar supplemented with 0, 5, 10, and 20 μg/ml vancomycin. The plate without any antibiotic served as the control to confirm the normal growth of bacteria. (**B**) MS_Rv1152 and MS_Vec were spotted on the 7H10 plate without any antibiotic after 24 hour treatment with 0, 5, 10, 20, 40, 80 μg/ml vancomycin, respectively. The bacteria numbers were counted after 3 days of incubation.

Vancomycin (Van), the last-resort antibiotics against infections caused by meticillin-resistant *Staphylococcus aureus* (MRSA)^34^, is a glycopeptide blocking the transpeptidation and nascent peptidoglycan synthesis. Van, in combination with lipid biosynthesis targeting antibiotic, was recently found more effective in killing multidrug-resistant (MDR) and extensively-drug resistant (XDR) *M. tuberculosis*^35^. Interestingly, Rv1152 decreased susceptibility of *M. smegmatis* to vancomycin, as the MIC of MS_Rv1152 for vancomycin is 80 μg/ml while MS_Vec is 20 μg/ml, there is no significant difference in MIC when using Cip, OFL, Nor, Ery, INH, and Rif (Table 3). In comparison with MS_Vec group, MS_Rv1152 is less susceptible to vancomycin even at the concentrations of 5 μg/ml. No bacteria of MS_Vec were detected on the plate supplemented with 20 μg/ml vancomycin (Fig. 3A). In addition, the obvious difference in survival of bacteria between MS_Rv1152 and MS_Vec were detected after treatment with vancomycin with various concentrations of for 24 h: there was about 1.3 × 10^5^ (CFU/ml) of MS_Rv1152 survived while no colony of MS_Vec can be detected when 80 μg/ml vancomycin was used (Fig. 3B). These data suggest that overexpression of Rv1152 in *M. smegmatis* contributes to reduced susceptibility to vancomycin.

### The isogenic deletion of MSMEG_5174 increased susceptibility to vancomycin

**Figure 4:**
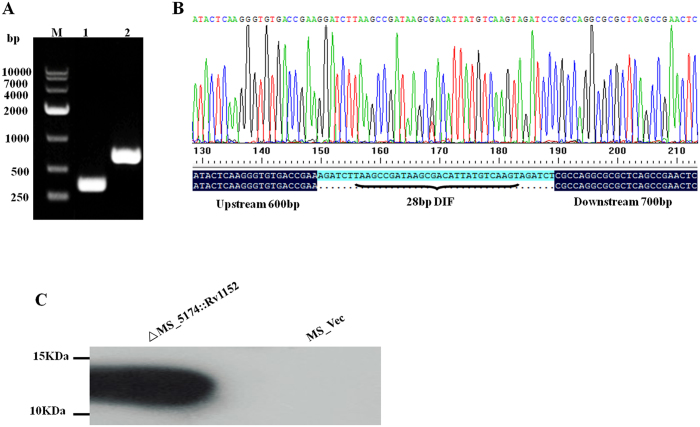
Construction of the △MSMEG_5174 and △MSMEG_5174::Rv1152 strains. (**A**) confirmation of MSMEG_5174 gene knockout by PCR amplification from WT and △MSMEG_5174 strains. M, Marker DL10000; 1, PCR band from △MSMEG_5174 strain; 2, PCR band from WT strain. (**B**) A map of the sequencing of PCR amplification products and DNAMAN alignment showing 28 bp DIF in △MSMEG_5174 strain. (**C**) Western blot showed that the Rv1152 gene was expressed in △MSMEG_5174 strain.

**Figure 5:**
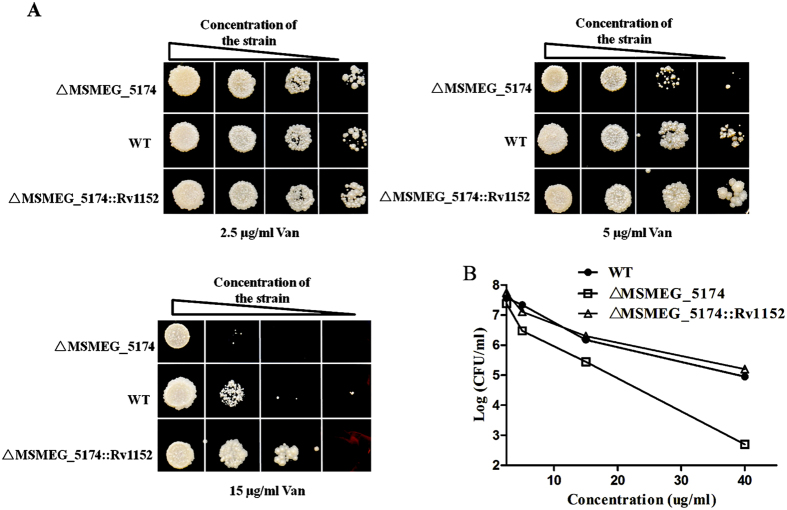
Susceptibility of △MSMEG_5174 strains to vancomycin. Ten-fold serial dilutions of wild type, △MSMEG_5174 and △MSMEG_5174::Rv1152 were plated on Middlebrook 7H10 with different concentration of vancomycin. Numbers of the bacteria were counted after 3 days incubation.

In order to further study the role of Rv1152 in vancomycin susceptibility, the homologous gene of Rv1152 in *M. smegmatis,* MSMEG_5174, was deleted (△MSMEG_5174). As shown in Fig. 4A, the sizes of the fragments amplified from the two colonies were consistent with expectations (300 bp for the mutant and 657 bp for the WT). The fragment amplified from the mutant bacteria was sequenced to verify the correctness of the excision: as expected, a copy of the DIF sequence flanked by two *Bgl*II restriction sites was found in the correct position, replacing the 247 central nucleotides of the target gene (Fig. 4B). Rv1152 was successfully expressed in △MSMEG_5174 after transformation Rv1152 gene into △MSMEG_5174 strain (Fig. 4C). The MSMEG_5174 gene knockout strain is more sensitive to vancomycin than the wild type *M. smegmatis*: most bacteria of △MSMEG_5174 were inhibited when using vancomycin at the concentration of 5 μg/ml and this sensitive phenotype was complemented in △MSMEG_5174::Rv1152 strain. No significant difference between WT and △MSMEG_5174 was detected when using other antibiotics (Table 4). The same result was reiterated when the strains were treated with different concentration of vancomycin (Fig. 5): with the increasing of the concentration of vancomycin, MSMEG_5174 deleted mutant are more sensitive than the WT strain, no bacteria of △MSMEG_5174 strains were detected, while there were obvious WT bacteria survived when using vancomycin at the concentration of 40 μg/ml.

### Rv1152 negatively regulates the expression of vancomycin responsive genes

**Figure 6:**
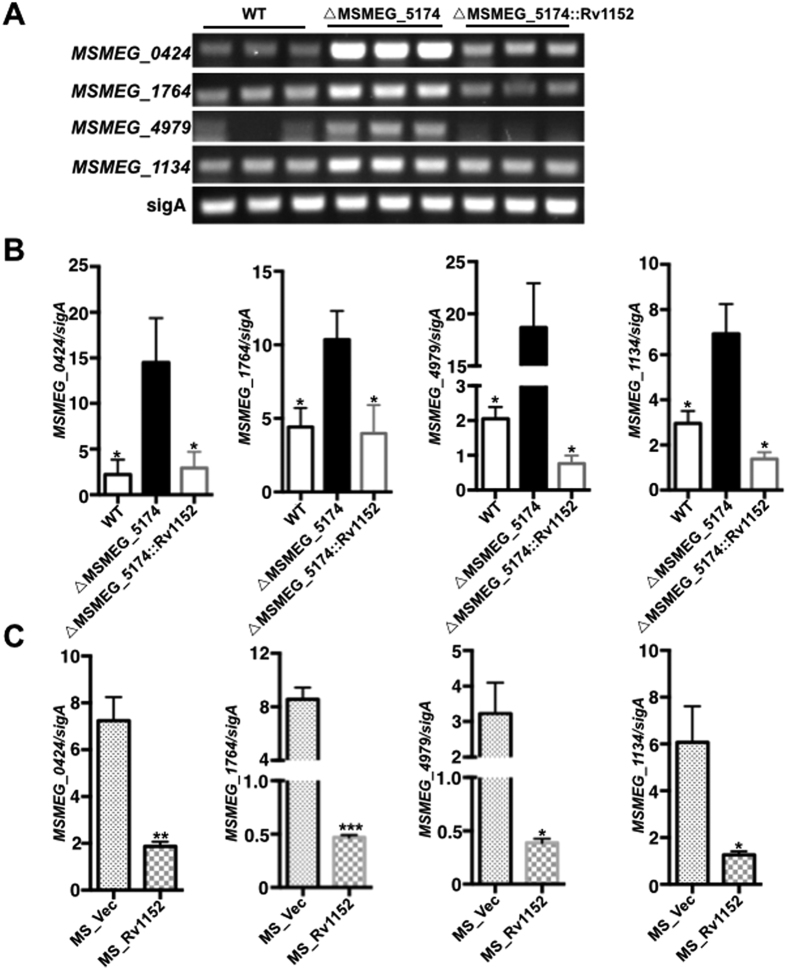
Transcription of genes responsive to vancomycin and regulated by Rv1152. (**A**) Semi-quantitative RT-PCR. (**B**) qRT-PCR assay of the relative transcription of target genes in WT, △MSMEG_5174 and △MSMEG_5174::Rv1152. (**C**) qRT-PCR assay the relative transcription of target genes in MS_Vec and MS_Rv1152 strains.

*M. smegmatis* has been widely used as a surrogate model bacterium to study the gene regulation and signal transduction mechanism of pathogenic mycobacteria^23^,^36^. The vancomycin responsive genes that transcriptionally regulated by Rv1152, together with their corresponding homologous genes in *M. smegmatis* were summarized in Table 5. The genome-wide regulator-DNA interaction network of *M. tuberculosis H37Rv* has shown that 68 genes are regulated by Rv1152^37^. Global transcriptome has shown that several *M. tuberculosis* genes are differentially regulated upon exposure to vancomycin^38^. To define the molecular mechanisms of the role of Rv1152 in vancomycin susceptibility, we tested whether vancomycin responsive genes are regulated by Rv1152. In comparison with WT strains, four genes including heat stress induced ribosome binding protein (MSMEG_0424), L-lysine-epsilon aminotransferase (MSMEG_1764), small unit of sulfate adenylyltransferase (MSMEG_4979), and protease transmembrane protein heat shock protein (MSMEG_1134) were up-regulated in △MSMEG_5174 strain, such expression pattern was restored in the Rv1152 complementary strain (Fig. 6A). The relative expression of these four genes to *sigA* was also up regulated after the homologous gene MSMEG_5174 in *M. smegmatis* was knock out (Fig. 6B), suggesting that Rv1152 negatively regulated the expression of these four genes. There was no significant difference for other genes listed in the Table 5 among WT, △MSMEG_5174 and △MSMEG_5174::Rv1152 strains (data not shown). We further identified Rv1152 inhibited the expression of these four target genes in *M. smegmatis* (Fig. 6C). These data suggest that Rv1152 represses the expression of the four vancomycin responsive genes.

### Rv1152 regulated genes affect susceptibility to vancomycin

**Figure 7:**
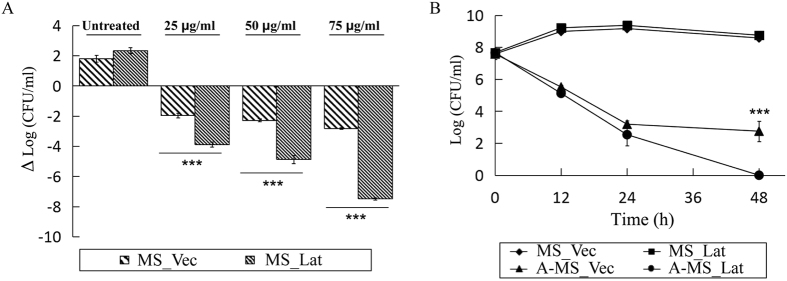
Overexpression of Lat in *M. smegmatis* sensitizes vancomycin susceptibility. (**A**) Log change in colony-forming units (CFU)/ml, from time zero, of MS_Vec (Log Cfu/ml = 7.2999) and MS_Lat (Log Cfu/ml = 7.4926) after treatment for 24 hours with different concentrations of vancomycin. (**B**) Killing curves of MS_Lat and MS_Vec upon 50 μg/ml vancomycin exposure for the indicated times. ♦untreated MS_Vec; ◾untreated MS_Lat; ▴antibiotic-treated MS_Vec; ⦁antibiotic-treated MS_Lat.

**Figure 8:**
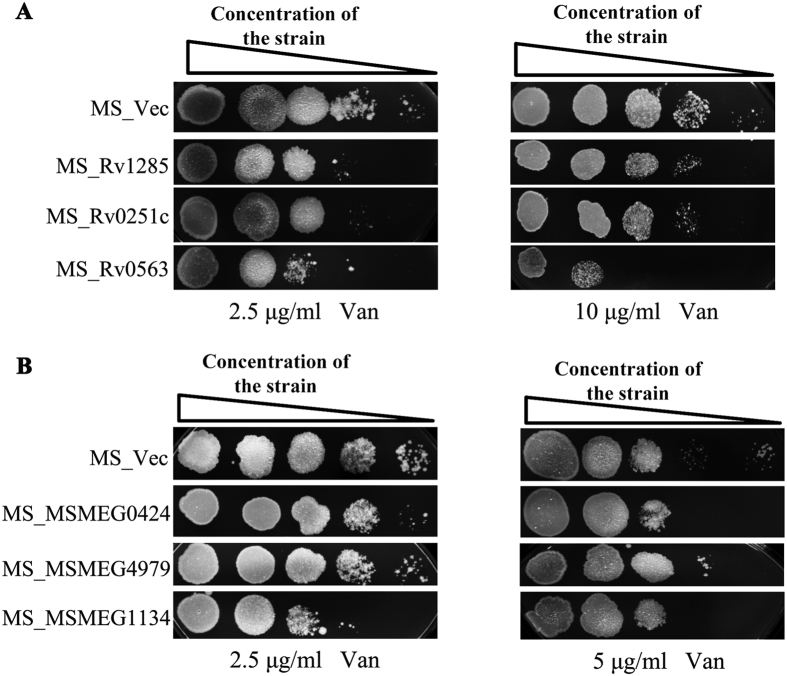
Overexpression of genes regulated by Rv1152 sensitizes vancomycin susceptibility. (**A**) Ten-fold serial dilutions of MS_Vec, MS_Rv1285, MS_Rv0251, MS_Rv0563 plated onto 7H10 agar medium supplemented with 2.5 and 10 μg/ml vancomycin, respectively. (**B**) Ten-fold serial dilutions of MS_Vec, MS_MSMEG_0424, MS_MSMEG_4979 and MS_MSMEG_1134 plated onto 7H10 agar medium supplemented with 2.5 or 5 μg/ml vancomycin, respectively. The plate without any antibiotic served as the control to confirm the normal growth of bacteria.

To determine whether Rv1152 regulated genes affect susceptibility to vancomycin, the recombinant strains expressing vancomycin responsive genes from *M. tuberculosis* and their homologous genes from *M. smegmatis* were constructed (Fig. 1S). The MIC for vancomycin of recombinant strains MS_Lat, MS_Rv1285, MS_Rv0251 and MS_Rv0563 was lower than MS_Vec, similar results were obtained from the *M. smegmatis* homologous gene recombinant strains (Table 2S). We have recently identified a lysine-epsilon aminotransferase (Lat) encoded by Rv3290c was involved in persister formation^39^. Interestingly, *M. smegmatis* overexpressing Lat exhibited more susceptibility to vancomycin than the wild type carrying the control vector –MS_Lat showed a greater bacterial cell death than MS_Vec (Fig. 7A). After 48 hour treatment with high concentration vancomycin, no bacteria was detectable (detection limit of 100 bacteria/ml) for MS_Lat while many colonies were recovered with MS_Vec control (Fig. 7B). These results demonstrate that overexpression of Lat in *M. smegmatis* make the host strain more sensitive to vancomycin. Overexpressing of another three vancomycin responsive genes (Rv_1285, Rv_0251, and Rv0563) from *M. tuberculosis* and their homologous genes (MSMEG_4979, MSMEG_0424, and MSMEG_1134) from *M. smegmatis* decreased susceptibility of host bacteria to vancomycin (Fig. 8). These data suggest that Rv1152 negatively regulated genes are responsive to vancomycin, resulting in decreased susceptibility of host bacteria to vancomycin.

## Discussion

The unusual ability of *M. tuberculosis* to persist for years within human host, and the requirement for lengthy antibiotic combination regimens to eliminate drug sensitive strains are hallmarks of this recalcitrant pathogen. Its prolonged chronic infection requires the expression of a complex array of genetic determinants, including those involved in secondary metabolism, cell wall processes, stress responses, and signal transduction^40^. Transcriptional regulators are well-established key players for the mycobacteria adaptation, such as two-component regulatory systems, stress-responsive sigma factors^41^ including *sigB, sigE,* and *sigH*^42^,^43^,^44^. We expressed GntR-like family transcriptional regulator Rv1152 in *M. smegmatis* and found that Rv1152 localized to both cell wall and cytoplasm. The up-regulation of Rv1152 after 96 h of starvation prompted us to test whether recombinant MS_Rv1152 will affect the permeability to these antimicrobial factor, the growth characteristics of MS_Vec and MS_Rv1152 under acid and surface stress were analyzed, as the transcription of *sigB* depends on *SigE* under physiological conditions and following exposure to SDS^41^. Our data identified that overexpression of Rv1152 in *M. smegmatis* has no effect on the bacterial growth in standard culture conditions (Fig. 2S), while Rv1152 can modify the response of *M. smegmatis* to different stresses. Rv1152 enhanced tolerance of *M. smegmatis* to SDS, but it sensitized bacteria to acid stress.

*Mycobacterial* responses to stress are of particular interest to understand the pathogenesis of *M. tuberculosis* and its sensitivity and reaction to antibiotics^33^. The detailed pathways, signals, regulatory responses and molecular interactions are not yet well understood, despite of the existence of 190 in silico predicted transcription regulators in the *M. tuberculosis* genome^45^. The genus *Mycobacterium* includes both pathogenic and saprophytic species that are able to survive under environmental stresses, including oxidative and genotoxic stress, hypoxia, nutrient starvation, and multiple antibiotics^7^,^8^. We suspected that Rv1152 might be involved in vancomycin susceptibility since the expression of Rv1152 was up-regulated under vancomycin exposure^38^. In congruent with our expectation, the expression of Rv1152 in *M. smegmatis* host bacteria loss of susceptibility to vancomycin. No difference was detected between MS_Vec and MS_Rv1152 in susceptibility to the other six antibiotics including norfloxacin, ciprofloxacin, ofloxacin, erythromycin, isoniazid, and rifampicin. To elucidate the molecular mechanism underlying Rv1152 vancomycin loss of susceptibility. The MSMEG_5174 knock-out strain (△MSMEG_5174) was constructed, MSMEG_5174, the *M. smegmatis* homolog of Rv1152, bearing the signature of the YtrA subfamily member^21^. In comparison with the parental strain, MSMEG_5174 gene knock out has little effect on the growth of *M. smegmatis* (Fig. 3S). △MSMEG_5174 is more susceptible to vancomycin than the parental *M. smegmatis*, suggesting the involvement of MSMEG_5174 in the tolerance to vancomycin. In addition, the response of Rv1152 complemented △MSMEG_5174 strain to vancomycin was restored to the level of parental *M. smegmatis*. Taken together, our data indicate that Rv1152 plays an important role in vancomycin loss of susceptibility.

*M. tuberculosis* transcriptome alteration in response to vancomycin^38^ was mined for genes regulated by Rv1152 and involved in vancomycin loss of susceptibility. We found that the expression of four vancomycin responsive genes were down-regulated in Rv1152 overexpression *M. smegmatis*, including ribosome binding protein (MSMEG_0424), small unit of sulfate adenylyltransferase (MSMEG_4979), L-lysine-epsilon aminotransferase (MSMEG_1764), and protease HtpX (MSMEG_1134), respectively. Their expression was also increased in response to vancomycin treatment. The expression of these four genes was increased in △MSMEG_5174, and the complementary strains △MSMEG_5174::Rv1152 showed the restored levels of transcription for these four genes. We further found these four genes are responsible for susceptibility to vancomycin. In summary, our data demonstrate that *M. tuberculosis* Rv1152 plays a role in vancomycin loss of susceptibility via negatively regulating the expression of genes responsive to vancomycin. Vancomycin is a robust glycopeptide antibiotic against multiple drug resistant clinical strains. This is the first report of *Mycobacteria* GntR family transcriptional factor involved in vancomycin loss of susceptibility. Further discovery of inhibitors against Rv1152 may provide good adjuvants for vancomycin or other antibiotics targeting the cell wall biosynthesis.

## Additional Information

**How to cite this article**: Zeng, J. *et al*. *Mycobacterium tuberculosis* Rv1152 is a Novel GntR Family Transcriptional Regulator Involved in Intrinsic Vancomycin Resistance and is a Potential Vancomycin Adjuvant Target. *Sci. Rep.* **6**, 28002; doi: 10.1038/srep28002 (2016).

## Supplementary Material

Supplementary Information

## Figures and Tables

**Table 1:** The list of strains and plasmids used in the study.

Strains	Description of strains	Source
WT	Wild type *M. smegmatis* mc^2^ 155 strain	
MS_Vec	*M. smegmatis* with transformed with vector pALACE	This study
MS_Rv1152	*M.smegmatis* with transformed with vector pALACE_Rv1152	This study
△MSMEG_5174	Parent strain *M. smegmatis* with an in-frame *MSMEG_5174* deletion mutation from the genome.	This study
△MSMEG_5174::Rv1152	*MSMEG_5174* mutate strain transformed with a inducible vector pALACE_Rv1152	This study
*E. coli* DH5a	Strain used in vector proliferation	Invitrogen
*E. coli*_Rv1152	Strain DH5a transformed with plasmid pALACE used for vector proliferation.	This study
Plasmids	Description of Plasmids	
pALACE	A replicative plasmid used for gene expression in *M. smegmatis* and conferring hygromycin (hyg) resistance	
pJV53	A replicative plasmid expressing two phage recombinases and conferring kanamycin (Km) resistance	

**Table 2:** Primers used in the study.

Primer	Description	Sequence of primers (5′-3′)
Rv1152-F1	Construct recombinant MS_Rv1152 strain	GGATCCCGCCAGGCGCGCTCAGCCG
Rv1152-R1		AAGCTTCCGAAGGCGACCGTGGTGG
KO-F1	Amplify MSMEG_5174 upstream sequence	TATGGATCCTACGTGGAGCTGCG
KO-R1		GTTGCATCGATTCAGTCGTCCGG
KO-F2	Amplify MSMEG_5174 downstream sequence	AAGCTTCGCTCGGGGTTGACCTCGA
KO-R2		TCGGCTGAGCGCGCCTGGCGGGATCCTTCGGTCACACCCTTGAGTATG
Rv1152-F2	Confirm dif sequence in mutation	AACGTGGGCACGCCGCTTT
Rv1152-R2		TGTCATATGTCAGTCCAGCGCGG
Lat-F1	Construct recombinant MS_Lat strain	TGCTTCATCGCCGAACCCATCCA
Lat-R1		CGAACGAGACCACATCGGGCATCACA
Rv0251c-F1	Construct recombinant MS_Rv0251c strain	GACGGATCCATGAACAATCTCGCA
Rv0251c-R1		CGCGCCATATGCTACTTCGTGAT
Rv1285-F1	Construct recombinant MS_1285 strain	CTGGTCGGATCCATGGCAATAAC
Rv1285-R1		CTGGTCGGATCCATGGCAATAAC
Rv0563-F1	Construct recombinant MS_0563 strain	GGAGGACACGGATCCATGACTTGGC
Rv0563-R1		GGAATTCCATATGTCAGCCGCGCGCCAT
MSMEG_0424-F1	Construct recombinant MSMEG_0424 overexpressing strain	AGGCGGATCCATGAGCACGCTGATGAA
MSMEG_0424-R1		GCGGGTTTCATATGTTACCGGCTCTCG
MSMEG_4979-F1	Construct MSMEG_4979 overexpressing strain	AACGGATCCATGACCGCAGTCGACAG
MSMEG_4979-R1		GCGTCGCCATATGTCAGAAATACCCC
MSMEG_1134-F1	Construct MSMEG_1134 overexpressing strain	GACGGATCCATGACGTGGAATCC
MSMEG_1134-R1		CGCCATATGTCAGTAGTACGAGTCC

**Table 3:** The response of MS_Vec and MS_Rv1152 to antibiotics.

Antibiotics (μg/ml)	MS_Vce	MS_Rv1152
Van	20	80
INH	8	8
Rif	16	16
Ery	64	64
Nor	32	16
OFL	0.25	0.25
CIP	0.25	0.25

**Table 4:** The response of WT, △MSMEG_5174 and △MSMEG_5174::Rv1152 to antibiotics.

Antibiotics (μg/ml)	WT	△MSMEG_5174	△MSMEG_5174::Rv1152
Van	20	5	20
INH	64	32	64
Rif	16	8	16
Ery	64	128	128
Nor	32	32	32
OFL	0.25	0.25	0.25
CIP	0.25	0.25	0.25

**Table 5:** The genes responsive to vancomycin.

Gene	Name	Homologous	Product
*Rv0824c*	desA1	MSMEG_5773	Probable acyl-[acyl-carrier protein] desaturase DesA1 (acyl-[ACP] desaturase) (stearoyl-ACP desaturase) (protein Des)
*Rv0251c*	hsp	MSMEG_0424	Heat shock protein Hsp (heat-stress-induced ribosome-binding protein A)
*Rv0516c*		MSMEG_0586	Possible anti-anti-sigma factor
*Rv0563*	htpX	MSMEG_1134	Probable protease transmembrane protein heat shock protein HtpX
*Rv2050*		MSMEG_3858	Conserved protein/ RNA-polymeraseassociated protein
*Rv2623*		MSMEG_3945	Universal stress protein family protein TB31.7
*Rv2745c*		MSMEG_2694	Transcriptional regulatory protein ClgR
*Rv1152*		MSMEG_5174	Regulatory protein GntR, HTH
*Rv3290c*	*lat*	MSMEG_1764	Lysine-ε aminotransferase
*Rv2688c*	*rv2688c*	MSMEG_1502	Antibiotic ABC transporter ATP-binding protein
*Rv3862c*	*whiB6*	MSMEG_0051	Transcriptional regulatory protein
*Rv1285*	*cysD*	MSMEG_4979	Sulfate adenylyltransferase subunit 2
*Rv1153c*		MSMEG_5173	O-methyltransferase, putative
*Rv1151c*		MSMEG_5175	NAD-dependent deacetylase
