# Supplementary material for: Mycobacterium tuberculosis Rv1152 is a Novel GntR Family Transcriptional Regulator Involved in Intrinsic Vancomycin Resistance and is a Potential Vancomycin Adjuvant Target
Source: Sci Rep. 2016 Jun 28;6:28002. doi: 10.1038/srep28002 (PMC4923875; doi:10.1038/srep28002)
Supplement: Supplementary Information [file srep28002-s1.doc]

***Mycobacterium tuberculosis* Rv1152 is a Novel GntR Family Transcriptional Regulator Involved in Intrinsic Vancomycin Resistance and is a Potential Vancomycin Adjuvant Target**

**Jie Zeng^1,#^, Wanyan Deng^1,#^, Wenmin Yang^1^, Hongping Luo^1^, Xiangke Duan^1^, Longxiang Xie^1^, Ping Li^1^, Rui Wang^1^, Tiwei Fu^1^, Abualgasim Elgaili Abdalla^1,2^, Jianping Xie**^1^,*

1. Institute of Modern Biopharmaceuticals, State Key Laboratory Breeding Base of Eco-Enviroment and Bio-Resource of the Three Gorges Area, Key Laboratory of Eco-environments in Three Gorges Reservoir Region, Ministry of Education, School of Life Sciences, Southwest University, Beibei, Chongqing 400715, China.

2. Department of Clinical Microbiology, College of Medical Laboratory Sciences, Omdurman Islamic University, Omdurman, Khartoum, Sudan

^﹟^These authors contributed equally to this work, co-first author

*Correspondence and requests for materials should be addressed to J.X.(email: georgex@swu.edu.cn)

**Table 1S. RT-PCR primers used in the study**

| **Rv number** | **Homologous gene of *M. tuberculosis* in *M. smegmatis*** | **Length/bp** | **primer** |
| --- | --- | --- | --- |
| Rv0824c | MSMEG_5773 | 254 | P1:ACCGACGATTACACCGACCTTCAGC  P2:ATCTCGCGGTGGTAGGAGGGCA |
| Rv0251c | MSMEG_0424 | 214 | P1:GCCGACGACTGGTTCAAGGGATT  P2:AGGAGCCGTAGCGGACCTCACTGTA |
| Rv0516c | MSMEG_0586 | 193 | P1:GTGCTCGATCTGAGTGGGGTGAACA  P2:CGAAGTGATGCAGAGCGTCGGGTAC |
| Rv0563 | MSMEG_1134 | 198 | P1:CGTGATGTTCCTTGCGGTGTTGT  P2:TTGGCGGTGTCGGAGATGTAGAG |
| Rv2050 | MSMEG_3858 | 218 | P1:TACGAGACCGACCGCAACCAT  P2:CGCTCCAACAGCATGTCCCAGT |
| Rv2623 | MSMEG_3945 | 188 | P1:TCAAAGAGGCCCGCAAGGTC  P2:CGCAGTGACCGAACCCAAGAGT |
| Rv2745c | MSMEG_2694 | 153 | P1:GCGACGCACTCGACGTACCACTTT  P2:ACACCTGCGGGATGACCACCTT |
| Rv1152 | MSMEG_5174 | 173 | P1:GATCCCGATAGCGACAAACCG  P2:GCAGCTTCGAGTTCCCGGTA |
| Rv3290c | MSMEG_1764 | 178 | P1:CTGAACAAGCCCAGCAACTCCGAC  P2:CCTCGTTGTGCCTGCTCTTCCA |
| Rv2688c | MSMEG_1502 | 182 | P1:CGGAATGGATTTCGCCGTAGG  P2:ACACACCGACGCGCTGGTAGTA |
| Rv3862c | MSMEG_0051 | 146 | P1:GCAAAGGTCATCTGCCGCGAAT  P2:GAACGAAGCTGCTTGAGCGCGA |
| Rv1285 | MSMEG_4979 | 166 | P1:CGAGGCCATCCACATCATCC  P2:CGATGACCTCTTCGAAGTTGTGG |
| Rv1153c | MSMEG_5173 | 194 | P1:CAGACCAGCTTCTACCGCCTCA  P2:GTGATGAACACGCCCTCGGAGT |
| Rv1151c | MSMEG_5175 | 181 | P1:ATCCGTCCCAGCGTGGTGTGGTT  P2:GGTTGACCTCGATGACGGGCTT |

**Table 2S**. The response of Rv1152 regulated genes to vancomycin

| **Bacterial**  **Strain No.** | **MIC (μg/ml)** |
| --- | --- |
|  | **Vancomycin** |
| MS_Vec | 20 |
| Ms_Lat | 5 |
| MS_Rv1285 | 5 |
| MS_Rv0251 | 10 |
| MS_Rv0563 | 5 |
| MS_MSMEG_1134 | 2.5 |
| MS_MSMEG_0424 | 10 |
| MS_MSMEG_4979 | 5 |


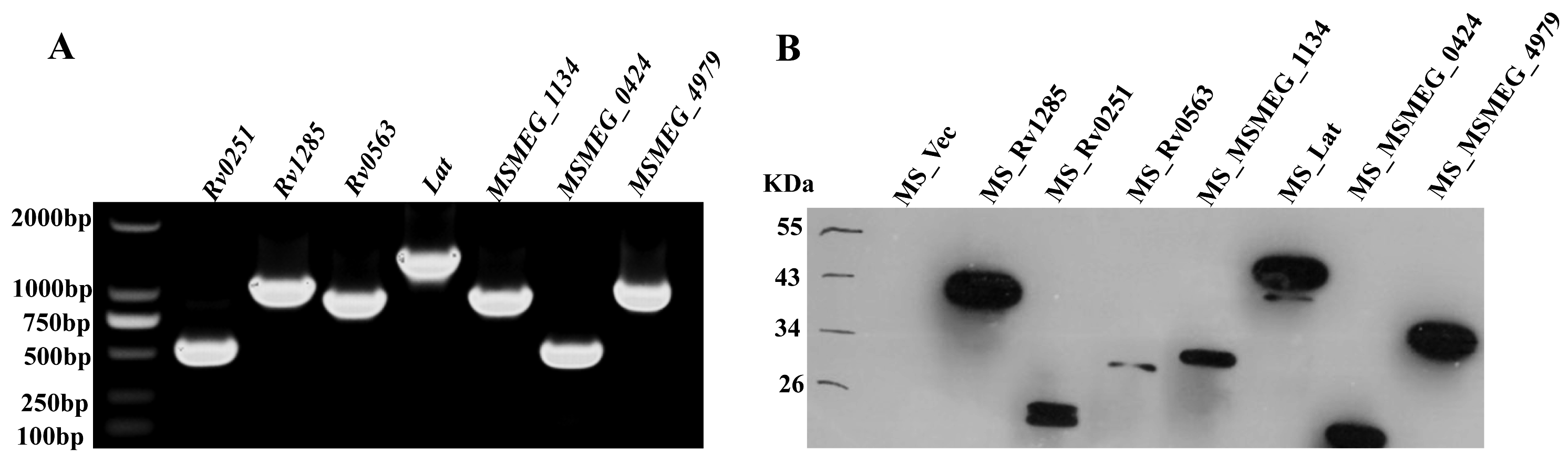


**Figure 1S.** Overexpression of Rv1152 regulated genes responded to vancomycin in *M. smegmatis*. (A) PCR amplification of Rv0251, Rv1285, Rv0563, Lat gene from *M. tuberculosis* and MSMEG_1134, MSMEG_0424 and MSMEG_4979 gene from *M. smegmatis*. (B) Western blot demonstrated the expression of His-tagged recombinant proteins in *M. smegmatis*. Lat encodes L-lysine-epsilon aminotransferase, Rv0251, heat shock protein Hsp, Rv1285, probable sulfate adenylyltransferase subunit CysD, Rv0563, probable protease transmembrane protein heat shock protein HtpX.


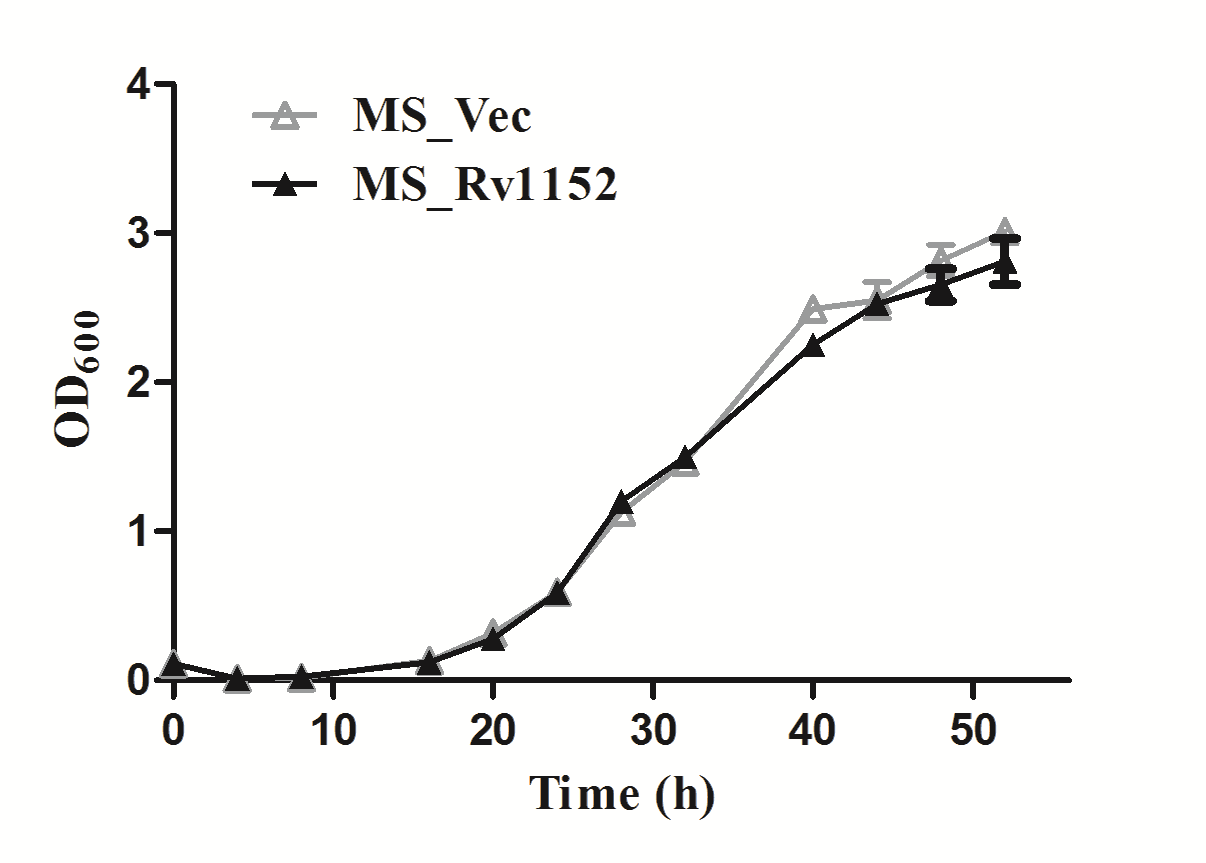


**Figure 2S.** Growth of recombinant MS_Vec and MS_Rv1152. Mycobacterium smegmatis MS_Vec and MS_Rv1152 were grown in Middlebrook 7H9 medium supplemented with 0.2% glycerinum and 0.05% Tween80. The OD_600_ were determined at an interval of 4 h.


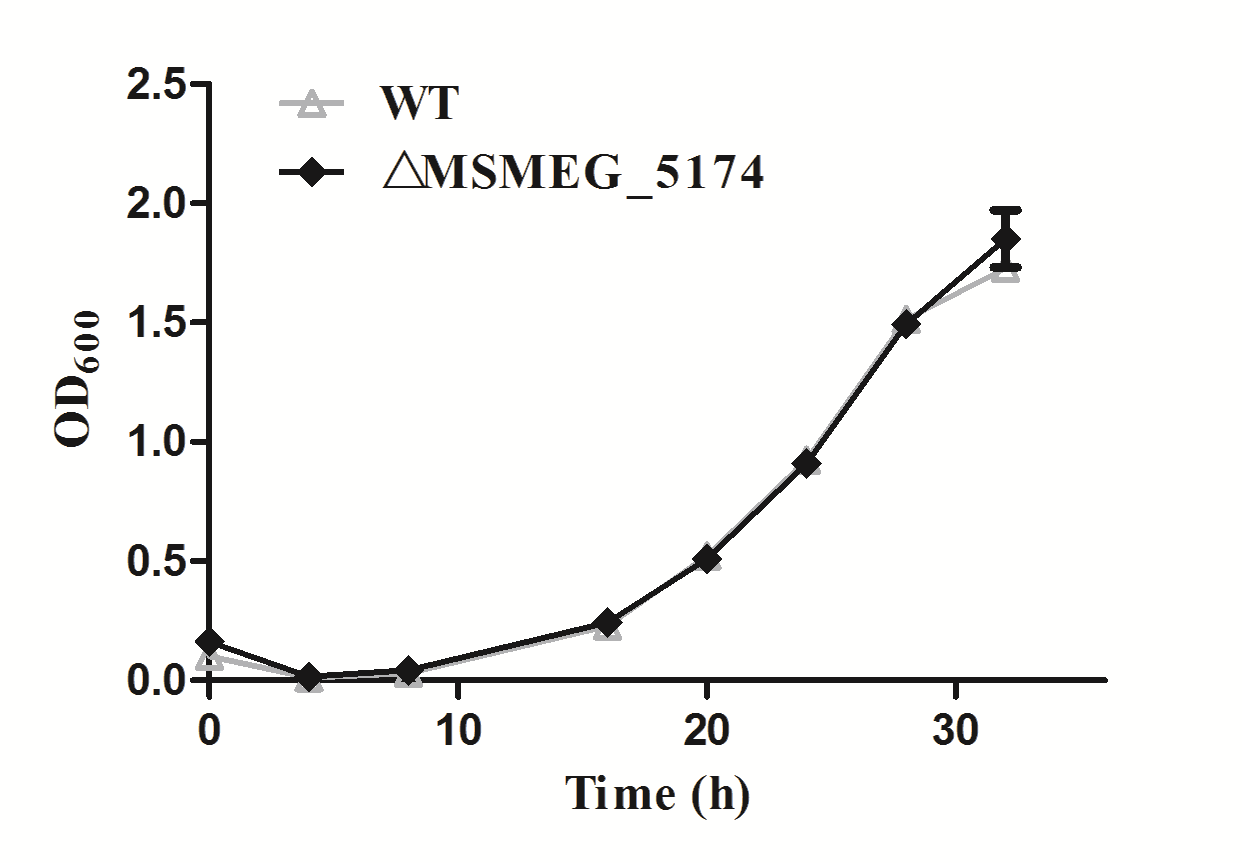


**Figure 3S.** Growth of WT and △MSMEG_5174. WT and △MSMEG_5174 were grown in Middlebrook 7H9 medium supplemented with 0.2% glycerinum and 0.05% Tween80. The OD_600_ were determined at an interval of 4 h.
